# Endoscope-Assisted Tonsillotomy (Partial Intracapsular Tonsillectomy): How We Do It

**DOI:** 10.5402/2012/713901

**Published:** 2012-04-19

**Authors:** Vincent Uzomefuna, Fergal Glynn

**Affiliations:** Otolaryngology Department, Our Lady's Children Hospital Crumlin, Dublin 12, Ireland

## Abstract

*Objective*. To describe our technique of performing tonsillotomy that increases visibility by providing a better view of the tonsils and related structures through the use of a 30-degree scope. *Method*. Patients had tonsillotomy with microdebrider with the aid of a 30-degree endoscope for both visualization and on-screen projection and magnification. *Result*. The endoscope-assisted technique provides a more detailed exposure of pharyngeal structures and their relationships with the tonsils. It is easier to clearly visualize the upper and lower poles. The magnification with the endoscope makes it easier to appreciate anatomic details and identify/deal selectively with minute bleeding points. *Conclusion*. The use of 30-degree endoscope in tonsillotomy provides better visualization of the upper and lower tonsil poles and may make the procedure easier for the surgeon and safer for the patient.

## 1. Introduction

Tonsillotomy was initially practiced in the 19th century and has been revived in the early 1990s [1]. After years of heated arguments among surgeons of the last century, tonsillotomy was eventually abandoned in favor of tonsillectomy as a result of changes in the understanding of the pathophysiology of some diseases such as rheumatism, scarlet fever, and chronic heart disease, which were thought to originate from the “diseased” remnants of tonsil tissue [2, 3]. The modern day tonsillotomy revival was originally aimed at reducing postoperative pain, but this was also found to significantly reduce the incidence of postoperative bleed [4].

**Figure 1:**
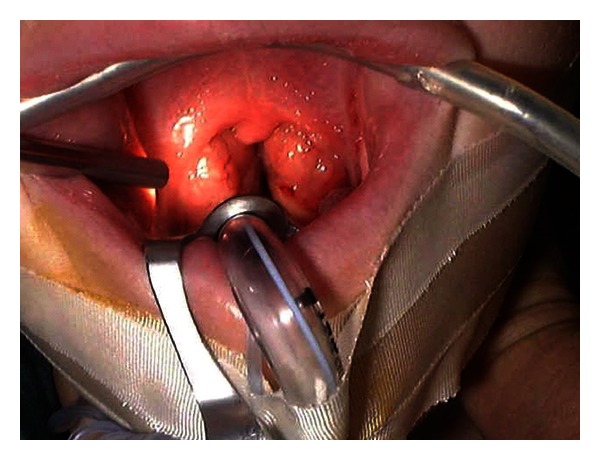
View without 30-degree scope.

Increasingly, tonsillotomy (partial intracapsular tonsillectomy) is being recommended over tonsillectomy in children < 4 years with tonsil hyperplasia or obstructive sleep apnoea, those with body weight < 15 kg and those with increased risk of bleeding, but technical difficulties with access, clear anatomical exposure, and visibility still remains, and it could often be quite problematic to visualize the tonsillar poles (Figure 1).

Vascular supply to the tonsils come mainly from the poles, and poor visibility in these areas could hamper the use of diathermy with precision during haemostasis.

## 2. Method/Procedure

In undertaking the endoscope-assisted technique, patient is placed on the supine position and the oral cavity splinted open with a Boyle-Davies gag as in standard tonsillectomy. The uvula is retracted anteriorly by means of a stitch on a clip. Dry gauze packing is applied to the inferior aspect of the oropharynx to limit blood flow towards the supraglottis.

**Figure 2:**
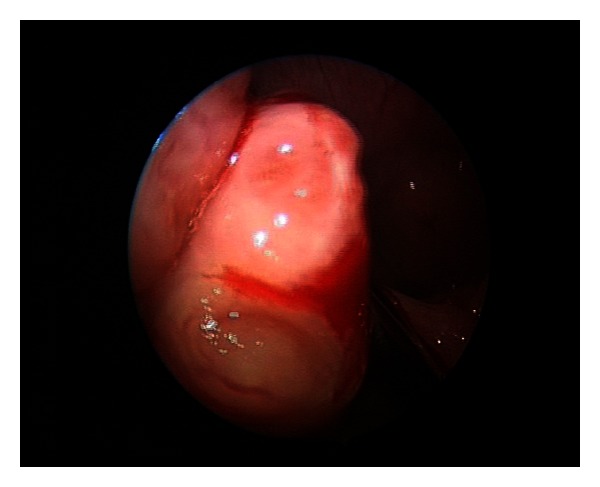
View of poles with 30-degree scope.

A 30-degree endoscope is introduced through the oral cavity, followed by a quick general inspection of the oropharynx and nasopharynx including the tonsils, adenoids, choanal aperture, and surrounding structures (Figure 2). 

The tonsils, tonsillar pillars, adenoids, choanae, and eustachian tubes are identified and inspected for any abnormality. The tonsils are then gently shaved down with a microdebrider. The aim is to shave off 90% of the tonsillar tissue without dissecting the tonsillar capsule from the adjacent muscles. 

**Figure 3:**
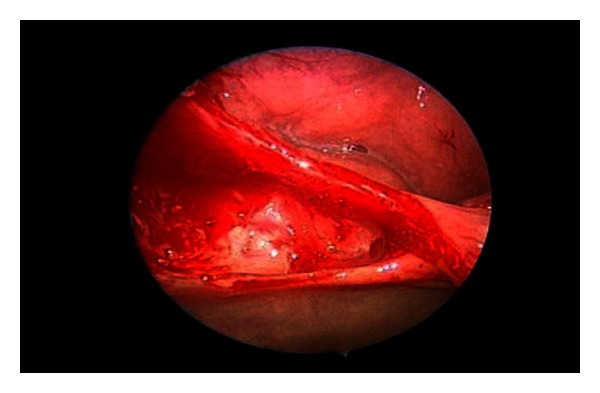
Posttonsillotomy view of upper pole with 30-degree scope.

Slight oozing of blood from the surface of the shaved tonsils is expected, and this can be controlled by applying adrenaline-soaked swab for a few minutes. The swab is then removed and the entire surface of the tonsil fossa including the upper and lower poles are examined for bleeding points using the endoscope, which in addition to making otherwise “blind spot” corners clearly visible also magnifies them (Figure 3).

The tonsil tissue itself is not very pain sensitive [5], and as the capsules of the tonsils are left intact, the nerve endings are spared and postoperative pain is either absent or very minimal. 

Performing tonsillotomy with minimal visibility of the upper poles (as is the case with tonsillotomy without scope assistance) is arguably the reason for various degree of breaching of the capsule, cutting into adjacent muscles and injuries to the palatal pillars; these inaccuracies explains the reason for presence of posttonsillotomy pain and increased risk of bleeding.

Not infrequently, the introduction of the endoscope reveals incidental findings of adenoid hypertrophy, and at the same instance, clearly shows the surgeon whether or not there is any submucous cleft palate that would contraindicate shaving the adenoids. Endoscopic adenoidectomy with necessary haemostasis under endoscopic guidance, as opposed to blind curettage of adenoid tissue, can then be carried out if indicated (endoscopic adenotonsillectomy). Most so-called adenoid regrowths after adenoidectomy are now known to be caused by inadequate removal of the adenoid tissues occasioned by fear of damage to surrounding structures during a blind curettage technique: these can all be avoided by direct visualisation “of what you are doing” using a 30 degree endoscope.

In our centre, the microdebrider is preferred to electrosurgical dissection as the heat generated from electrosurgical devices may be as high as 400°C, which can create a significant sphere of injury to the surrounding musculature of the tonsillar fossa [6]. This, in turn, may intensify the inflammatory process, contribute significantly to the amount of postoperative pain, and prolong the recovery period [7, 8].

Entire procedure is brief and, depending on the experience of the surgeon, could last between 5 and 15 minutes.

Pain is very minimal and patient is able to feed orally straightaway and could be discharged same day.

## 3. Conclusions

Tonsillotomy is an established method for reducing postoperative pain and bleeding, but still faced with challenges of visibility.

Visibility provided by the use of endoscope-assisted technique improves ease and operative precision.

Operative precision is vital in preserving the tonsil capsule, adjacent pharyngeal musculature, and the nerve endings.

Pharyngeal musculature, and nerve endings, when preserved, significantly reduce or eliminates postoperative pain and bleeding.
